# Addressing Smoking Cessation among Women in Substance Use Treatment: A Qualitative Approach to Guiding Tailored Interventions

**DOI:** 10.3390/ijerph18115764

**Published:** 2021-05-27

**Authors:** Isabel Martinez Leal, Matthew Taing, Virmarie Correa-Fernández, Ezemenari M. Obasi, Bryce Kyburz, Kathy Le, Litty Koshy, Tzuan A. Chen, Teresa Williams, Kathleen Casey, Daniel P. O’Connor, Lorraine R. Reitzel

**Affiliations:** 1 Department of Psychological, Health & Learning Sciences, The University of Houston, 3657 Cullen Blvd Stephen Power Farish Hall, Houston, TX 77204, USA; mtaing@central.uh.edu (M.T.); vcorreaf@central.uh.edu (V.C.-F.); emobasi@uh.edu (E.M.O.); lek1@livemail.uthscsa.edu (K.L.); lkoshy@central.uh.edu (L.K.); tchen3@central.uh.edu (T.A.C.); Lrreitze@central.uh.edu (L.R.R.); 2 Health Research Institute, The University of Houston, 4849 Calhoun Rd., Houston, TX 77204, USA; dpoconno@central.uh.edu; 3 Department of Health & Human Performance, The University of Houston, 3875 Holman Street, Garrison Gymnasium, Room 104, Houston, TX 77204, USA; 4 Integral Care, Austin, TX 78704, USA; bryce.kyburz@integralcare.org (B.K.); teresa.williams@integralcare.org (T.W.); kathleen.casey@integralcare.org (K.C.); 5 Long School of Medicine, The University of Texas Health Science Center at San Antonio, San Antonio, TX 78229, USA

**Keywords:** women-tailored smoking interventions, intersectionality, health disparities, polysubstance use, tobacco-free workplace

## Abstract

Intersecting socially marginalized identities and unique biopsychosocial factors place women with substance use disorders (SUDs) experiencing myriad disadvantages at higher risk for smoking and stigmatization. Here, based on our work with women receiving care for SUDs in four participating treatment/women-serving centers (N = 6 individual clinics), we: (1) describe the functions of smoking for women with SUDs; and (2) explore participants’ experiences of a comprehensive tobacco-free workplace (TFW) program, Taking Texas Tobacco-Free (TTTF), that was implemented during their SUD treatment. Ultimately, information gleaned was intended to inform the development of women-tailored tobacco interventions. Data collection occurred pre- and post-TTTF implementation and entailed conducting client (7) and clinician (5) focus groups. Using thematic analysis, we identified four main themes: “the social context of smoking,” “challenges to finding support and better coping methods,” “addressing underlying conditions: building inner and outer supportive environments,” and “sustaining support: TFW program experiences.” Women reported that: smoking served as a “coping mechanism” for stress and facilitated socialization; stigmatization hindered quitting; non-stigmatizing counseling cessation support provided alternative coping strategies; and, with clinicians, the cessation opportunities TTTF presented are valuable. Clinicians reported organizational support, or lack thereof, and tobacco-related misconceptions as the main facilitator/barriers to treating tobacco addiction. Effective tobacco cessation interventions for women with SUDs should be informed by, and tailored to, their gendered experiences, needs, and recommendations. Participants recommended replacing smoking with healthy stress alleviating strategies; the importance of adopting non-judgmental, supportive, cessation interventions; and the support of TFW programs and nicotine replacement therapy to aid in quitting.

## 1. Introduction

Although the rate of cigarette smoking among adults in the United States (US) has declined since 1965, reaching an all-time low rate of 13.7%, smoking remains the leading cause of preventable morbidity and mortality [1] and has been shown to disparately impact subgroups experiencing disadvantage (e.g., women); being recognized as a social justice issue affecting public health [2,3]. Disadvantaged subgroups include those experiencing social inequities and discrimination based social categories, e.g., gender, race/ethnicity, Indigeneity, class, sexuality, and ability. In the general population, men have historically smoked at higher rates than women, but this gender gap favoring women is shrinking. Recent studies have found that the rate at which women smoke has not declined as rapidly as their male counterparts, largely as a result of biopsychosocial differences [4,5,6,7,8,9,10,11]. In particular, these differences contribute to more severe impacts of smoking on women, increased difficulty with quitting smoking, and increased relapse following quit attempts, especially among specific subgroups [5,6,7,8,9,12]. 

More specifically, women facing multiple vulnerabilities, such as those with less education, living under socioeconomic disadvantages, and being diagnosed with mental health and/or substance use disorders (SUDs), are at increased risk for tobacco use and are less likely to successfully quit smoking [13]. For example, smoking rates among women using alcohol and other substances have been reported to range from 53.5% to as high as 71.7% [14]; these percentages may be greater among women in residential substance use treatment programs [15]. Additionally, compared to their male counterparts, women with SUDs face greater challenges addressing tobacco dependence due to higher lifetime rates of mood and anxiety disorders [16]. Women also face additional, gender-specific obstacles in accessing treatment for SUDs, including social stigma and child-caregiving pressures [17]. Unfortunately, researchers have documented a research-to-practice gap in adopting evidence-based practices for treating tobacco in substance use treatment centers (SUTCs) due to a widespread culture of smoking, high smoking rates among staff, misconceptions regarding clients’ desire to quit, and the idea that quitting might interfere with SUD recovery [18,19,20]. Research refutes these misconceptions, indicating that on the contrary, SUTC clients want to quit smoking [21], and that quitting can improve psychiatric symptoms [22,23] and SUD recovery by increasing abstinence from alcohol and other substances by 25% [18], and can increase psychological quality of life [24]. Sadly, more individuals with SUDs (>50%) die from smoking-related than from substance-related disease [25]. Moreover, women who have experienced trauma, domestic violence, and/or abuse are also at increased risk for tobacco and substance use [13,26,27]. Among women with posttraumatic stress disorder, recent studies indicate smoking rates ranged from 39.2% to 53.6% [26]. Taken together, these disparities, complemented by the intersecting challenges and stigmatization that female smokers experiencing disadvantage face, require tailored interventions to address the complex, multi-leveled factors influencing smoking behaviors among women in SUTCs [5,6,7,28,29]. An example of such a successful, evidence-based tobacco control intervention that uses a tailored approach is our Taking Texas Tobacco-Free (TTTF) program (www.takingtexastobaccofree.com) (accessed on 3 March 2021).

### 1.1. Taking Texas Tobacco-Free

TTTF is a multi-component tobacco-free workplace (TFW) program that adopts a center-specific approach to implementation designed to target known implementation barriers to comprehensive and sustainable tobacco cessation policies and practices. TTTF applies a system-wide strategy, seeking to ultimately affect a change in organizational culture regarding the treatment of tobacco addiction through changing tobacco use norms using strategies that reduce tobacco-related inequities among groups experiencing disadvantage (i.e., single mothers, those with substance use and mental health disorders, sexual minorities, those experiencing homelessness, former prisoners, and those of lower socioeconomic status). The means used to change smoking norms is through education and training of clinicians—and through them, clients—by correcting misconceptions regarding: (a) tobacco use as an effective “coping mechanism” for stress; (b) the effects of quitting smoking on SUD recovery; (c) the harms and prevalence of smoking; (d) the motivation and capacity of those with SUDs to quit smoking; and (e) the distortions and manipulations of the tobacco industry’s targeting of groups experiencing disadvantage. The program consists of: (1) adoption of organization-wide tobacco-free policies; (2) staff education on the harms of tobacco dependence; (3) specialized clinician training on how to assess and treat tobacco dependence using evidence-based interventions, i.e., tobacco-use screenings, behavioral interventions including the 5A’s and motivational interviewing, and FDA-approved medications and nicotine replacement therapies (NRT); (4) provision of free NRT and practical, hands-on guidance from the TTTF team throughout the implementation process; and (5) community engagement and outreach. Our comprehensive program focuses on treating tobacco dependence among groups experiencing disadvantage with the highest smoking rates and need for assistance in quitting. To date, TTTF has been successfully implemented in 300 mental health centers [30,31,32,33,34,35] and has recently expanded into 30 standalone SUTCs/community centers [36,37,38], four of which serve women only and are the subject of this report.

### 1.2. Theoretical Framework and Study Aim

Since Graham’s seminal work on the subject [39], researchers have shown a clear relationship between smoking and social and economic disadvantage among women [40,41]. Despite repeated calls for tailored interventions to address smoking cessation among women experiencing social and health inequities, few such programs have been developed [7,13,29,42]. Likewise, scant research has focused on how intersecting vulnerabilities influence women’s smoking [43,44]. Intersectionality is a theoretical framework that centers on understanding how multiple social identities (gender, class, race/ethnicity, behavioral health status, single motherhood) intersect in an interactive, rather than an additive, manner to [re]produce relationships of power and oppression [45,46]. The women within SUTCs/women-serving centers are especially vulnerable and at extremely high risk for smoking due to multiple, intersecting axes of inequality and identity: low socioeconomic status, low level of education, history of trauma and/or domestic violence, high stress, previous history in the criminal justice system, single motherhood, and having substance use and/or mental/behavioral health disorders [47]. As each of these is a stigmatized social category, an intersectional perspective can help elucidate how smoking—a stigmatized activity that can compound social isolation and stigmatization—is experienced by women living with multi-layered social inequities. 

Although this study primarily focuses on women with SUDs, who are also in the aforementioned groups experiencing vulnerabilities, it is important to note that the majority of women participating in this study were of childbearing age; most were young mothers and/or had small children, and a few were pregnant. Given the disparities in prenatal and postpartum smoking rates among low-income women and the significant risks associated with smoking during pregnancy, this study seeks to specifically examine the experiences of women smokers of childbearing age experiencing vulnerabilities [48,49]. To effectively address smoking cessation among this group of smokers, an understanding of the meanings of smoking in the lives of these women, as well as their needs and preferences regarding smoking interventions, must first be understood [39]. As smoking is recognized as a health inequity issue disproportionately borne by groups experiencing disadvantage globally, an intersectional perspective can assist researchers in being particularly sensitive to how tobacco control policies may exacerbate social justice and stigmatization issues among these groups [3]. This study fills a gap in the literature regarding the tailoring of smoking cessation interventions to the social context and needs of women with SUDs experiencing multiple disadvantages.

The current work describes an exploratory qualitative study, guided by an intersectional theoretical framework, to inform the adaptation of TTTF to the needs of women experiencing social and economic vulnerabilities who were seeking assistance at SUTCs and women’s centers. Study participants include both the women seeking assistance and the clinicians providing their care at three non-profit Texas SUTCs serving women only (at four locations), and one non-profit community center serving women only (at two locations), many of whom struggled with SUDs. The aims of this study were to understand participants’ perspectives on: (1) the meanings and functions of smoking within clients’ lives; and (2) participants’ experiences and recommendations regarding the TTTF program to identify what these women need to quit smoking and to inform the development of women-tailored tobacco interventions. 

## 2. Materials and Methods

### 2.1. Ethical Approval

This research was approved by the Internal Review Board of the University of Houston (STUDY00000472, approval date 27 July 2017). The nature of the study and interviews were discussed with participants who consented orally and were given the option of receiving a written consent document, prior to participation. Given the nature of group interviews, confidentiality could not be assured. However, participants were given the option of remaining anonymous or allowing the use of their names in the reporting of findings. Participants were also informed that their participation was entirely voluntary; they could decline to answer any questions and could withdraw from the study at any time. Permission for audio-recording of all interviews was granted prior to participation.

### 2.2. Design

This qualitative study is part of a larger mixed methods project focused on adapting and implementing a comprehensive TFW program, TTTF, within SUTCs and their affiliated community centers. Our aim to understand how participants construct their experiences and meanings around smoking and how those understandings can inform tailoring of women-centered cessation interventions guided the study design. A qualitative research design based on an intersectional [46] and social constructionist framework was chosen as most appropriate for the present study as this perspective focuses on the way in which women’s smoking is shaped by multiple identities and how they socially construct the world of experience and make sense or meaning of it [50]. Qualitative methodologies have been recognized as best suited to capturing and understanding individuals’ experiences and perspectives [51]. Client focus groups were used to explore women’s experiences with smoking and views on what they needed to successfully quit. Clinician focus group were also used to understand organizational culture, and what types of client support and services were being offered, both generally and those specific to tobacco control.

Different types of purposive sampling were used based on the size of the participating center. Criterion sampling was used with smaller centers, which involved selecting clients with smoking experience and clinicians involved with TTTF’s implementation. If the center was large enough to allow for additional variation in sampling, heterogeneous sampling was used in which program supporters as well as opponents were selected to capture a wide range of responses. The combination of different purposive sampling strategies increased the breadth and variation of data collection, and strengthened the analytical approach of the study [51]. Inclusion criteria included adults age 18 or older (for clients, those self-identifying as women, and who spoke English), and were either clients or clinicians providing direct client services (e.g., counselors, peer support) at a women-only serving SUTC or community center participating in the TTTF program (grant #PP170070). Focus group participants were recruited via coordination with the center program champion—a volunteer clinician or manager who was trained as a tobacco treatment specialist [52] as part of the TTTF program, and who was not additionally compensated for this position. 

### 2.3. Data Collection

Semi-structured interview guides were used to conduct focus groups with clients, and separately, with clinicians from October 2018 to October 2019, which consisted of 6–10 participants in each group; an additional individual clinician interview was conducted in October 2020. In-person focus groups, lasting 60–120 min, were conducted on-site at the various centers throughout Texas by a cultural anthropologist and public health practitioner (IML) trained in qualitative research. Following the outbreak of COVID-19, the individual clinician interview took place via a recorded videoconferencing platform [53]. Research aims guided the development of both interview guides, which remained open to change and were field-tested and revised based on responses in the field [51]. 

Focus groups were conducted at two time points, pre- and post- the TTTF implementation with each group. A pre/post design was selected so that data from the pre-implementation focus groups with clients and staff could guide the tailoring of the TTTF program to the specific needs, clients, and context of individual centers prior to implementation. For example, client and staff pre-implementation focus groups included reviewing program materials, (e.g., educational programs, brochures, posters) to elicit feedback used to tailor, and/or create additional materials to fit the center’s and clients’ needs (according to age, gender, sexual orientation, race/ethnicity, language); and additional questions on what clients needed to quit smoking, clients’ and staffs’ attitudes towards TFW programs, and center staffs’ attitudes towards smoking. These questions focused on better understanding the context of the center—characteristics, attitudes, populations, potential program obstacles and facilitators, and center organizational culture—to inform the design and adaptation of TTTF program materials and interventions to center needs.

Both pre- and post-implementation client focus groups focused on past/present smoking history, functions of smoking within daily life, perceptions of smoking, quit attempts, attitudes towards smoking and quitting, services (smoking-related and otherwise) received at the center, and connections between smoking and substance use. Additional client post-implementation questions included changes in center attitudes and smoking cessation services offered, experiences of quitting smoking during recovery, program experiences, and suggested program improvements and recommendations on what women needed to quit. Clinician pre-implementation focus groups included questions focused on general and specific tobacco-related support and services offered, expected challenges and facilitators to implementing tobacco cessation and unique center needs or characteristics regarding implementing a TFW program. Clinician post-implementation interviews included added questions on integrating the tobacco control program into center culture, implementation challenges and facilitators experienced, program adaptations, and suggested program improvements. Clients were compensated with a $10 gift card per focus group. In addition to audio-recording interviews, the interviewer also kept written notes that provided additional contextual information regarding interactions with participants.

### 2.4. Participating Centers

See Table 1 for characteristics of participating women-only serving centers. These centers provided services to women in 4 different cities. Most clinicians provided full-time treatment to clients, while others were engaged in outreach services to clients. 

Table 2 displays the intersecting categories of social inequity (unemployment, housing status, criminal justice status, history of violence/abuse or trauma, substance use and/or mental health disorder status, socioeconomic status) experienced by women receiving services at participating centers, as reported by center leadership. This table underscores how these women’s lives are embedded within, and constrained by, multidimensional, intersecting social inequalities.

### 2.5. Participating Clients and Clinicians

Overall, 7 focus groups were conducted with 59 clients across the 4 centers. Almost all clients who had SUDs had a long history of smoking. Most clients were current smokers, a few were former smokers, and a few had tried smoking but did not take up the habit, and only one had never smoked a cigarette before. Five clinician focus groups were completed with 23 clinicians who either were licensed or peer support counselors, and one individual clinician interview; there were 83 participants in total.

### 2.6. Data Analysis

All focus groups and interviews were transcribed verbatim by the researchers and uploaded onto Atlas.ti 8 (Atlas.ti, version 8.4, 2019) with field notes to organize and facilitate data analysis. Thematic analysis and constant comparison, based on an inductive approach, was used to systematically code raw qualitative data into patterns and themes [54]. While this analysis also drew on an intersectional and constructivist perspective, focusing on how participants position and construct themselves within social contexts, we used a data-driven approach, in which codes were drawn directly from the data rather than being predetermined in advance by theoretical frameworks. Data from the pre-implementation focus groups with both groups of stakeholders were analyzed first and compared across groups. Findings were used to understand women’s experiences of smoking and to adapt the TTTF intervention features to the local context. Post-implementation focus group data from both stakeholder groups were analyzed and compared to pre-implementation focus group data to understand any changes in participants’ attitudes towards smoking and the TTTF program, and staff attitudes towards treating tobacco dependence post-implementation.

The coding process started with familiarization with the data, reading and re-reading transcripts various times to identify and note recurring concepts. Coding progressed iteratively, using constant comparison, an ongoing process wherein emerging data are compared within and across previously coded transcripts, to condense codes into categories and themes drawn directly from the data. The first two authors (IML and MT), both trained in qualitative analysis, each independently coded 6 initial transcripts to develop a preliminary coding frame, then met to discuss and reconcile any coding discrepancies to refine the coding frame. The coding frame was revised three times to arrive at a final coding frame that was reapplied to all the data. The coding frame remained open throughout data analysis to develop and refine themes. The constant comparison process was used to refine codes, check for redundancy, ensure appropriateness of categories and themes, accurately account for all the data, and fulfilment of data saturation [55]—the point at which no new codes are found in the data. To respect participant privacy and confidentiality, pseudonyms are used throughout this article.

## 3. Results

**Figure 1:**
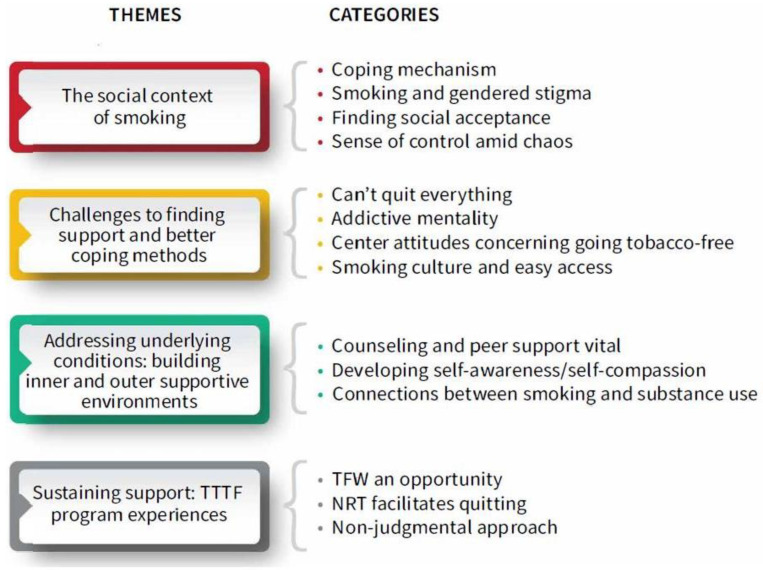
The meanings of smoking and women’s needs for quitting.

As an inductive approach to analysis was used, themes were drawn directly from the raw data reported by women rather than being derived a priori, with the exception of the theme regarding participants’ experiences of TTTF was deductively arrived at, being based on questions regarding program experiences, although the codes and categories within this theme were not predetermined. Themes reflect women’s concerns about the meanings of smoking in their lives, their desires and challenges regarding quitting, their insights on what they need to support them in quitting, and their experiences of a TFW program, TTTF, tailored to their suggestions. Substantial quotes are offered as evidence in support of analytic findings. Analysis of transcripts from focus groups with clients and, separately, clinicians resulted in 4 main themes: (1) the social context of smoking, focused on women’s perceptions of smoking and its role in their lives; (2) challenges to finding support and better coping methods, focused on women’s perspectives on what reinforces their smoking, and organizational and clinician attitudes and factors hindering quitting smoking; (3) addressing underlying conditions: building inner and outer supportive environments, encompassed women’s and clinician’s suggestions on what women need to best support them in quitting smoking; and (4) sustained support: TTTF program experiences. Figure 1 displays the 4 themes and their categories. 

In addition to the findings from the thematic analysis, we separately report women’s and clinicians’ recommendations that were used to tailor the TTTF program to women’s smoking cessation needs and preferences.

### 3.1. The Social Context of Smoking

#### 3.1.1. “Calms Me Down”/Coping Mechanism

Most women reported that smoking was a “coping mechanism”, that it “calms me down,” “relieves stress,” or “numbs me” so they could deal with the stress of living under the burden of intersecting social and economic inequities. Women described experiencing smoking as a means of coping with or easing feeling overwhelmed, angry, upset, anxious, or depressed. They related craving a cigarette most when they were feeling distressed, irritated, or wanted to relax. Many women related living with trauma, “*Everyone here is traumatized*” (Helen, SUTC1), and smoking to cope with overwhelming feelings:

*I’d smoke a cigarette to calm myself down and I feel it was a pacifying thing… when you’re struggling with stuff that you’ve experienced… a lot of people that smoke have underlying issues. You don’t just drink or smoke for no reason, you’re trying to get away from something that’s hurting you, you’re trying to escape something that’s painful… so it’s deeper.*

(Rose, Women’s Center 1)

Some women related that smoking was an effective coping mechanism and did not seem to perceive it as an addiction. However, they spoke about smoking in the same way they spoke about the other substances they were addicted to, in terms of needing the drug to alleviate distress or discomfort of some sort. Moreover, paradoxically, as with other addictions, Rose, who is quoted above regarding the calming effects of smoking, also saw it as a self-harming and unhealthy behavior:

*I noticed I could quit cigarettes really easily, but it was never a matter of whether I could quit, but more like I felt I needed it when I was stressed out. Like it’s not an addiction so much as I need to get rid of the stress and the anxiety… I started with a lot of self-loathing and I purposely hurt myself in different ways even subconsciously, like with smoking.*

(Rose, Women’s Center 1)

#### 3.1.2. Smoking and Gendered Stigma

Many single mothers stated that they felt overwhelmed with the demands of caring for often multiple children by themselves. For these single mothers, smoking provided a temporary, but to them, necessary respite from the stress of caring for young children:

*When my kids are getting on my nerves—I have 3 children all under the age of 5, okay—it’s probably a good thing that I can take those 5 min to smoke a cigarette and just—‘Whew’—catch a break, you know?*

(Mary, Women’s Center 2)

However, regarding children, smoking was also experienced as stigma, and a source of shame; most women said they would not smoke in front of their children and most reported feeling ashamed about smoking while they were pregnant. During a focus group, women who were pregnant were shamed by other mothers for continuing to smoke:

*You’re still smoking while you’re pregnant? [incredulous] I quit when I was pregnant…You need to quit because it’s bad for your baby. [Others present agree]*

(Pat, SUTC1)

Many women’s primary reason to quit smoking was for the health of their children. The only time many had quit smoking was while they were pregnant, during up to 4 successive pregnancies, although some eventually resumed smoking. Even so, many felt shame for smoking, as their children had asked them to quit:

*I feel like one of the most negative things for me is the way my kids react to it. It eats at me because my kids will say, ‘Mom you’re smoking.’ My 3-year-old will be like, ‘Mom put that cigarette down that’s bad for you’… I want to quit, I need to quit, but it’s so hard*.

(Grace, Women’s Center 2)

#### 3.1.3. Developing Social Acceptance 

Women’s experiences of smoking were fraught with other contradictions. As with most addictions, they wanted to quit, and expressed hating and loving smoking:

*Yes, I want to quit. There is a side of me that’s just like grotesqued about it, you know? And there’s another side of me that still really wants it.*

(Alma, SUTC1)

Despite feeling stigmatized for smoking as single mothers, women also reported deriving pleasure from it as a social activity. Most women had initiated smoking in their early teens to gain social acceptance within their social networks of friends and family. Likewise, as the women’s center—and prior to implementing TTTF, all but one of the SUTCs—allowed smoking in designated areas, smoking inadvertently became the primary social activity for women. Clinicians and clients reported that women who were non-smokers upon entry to the program took up—and some who had quit, resumed—smoking, which was described as the currency paid for belonging socially:

*Smoking is how I made friends here, we hung out because we all smoked. Now that I don’t smoke, it makes it really hard to be out there because they’re all still smoking and I’m just standing there wanting one. That’s one of the hardest things is that even when you do quit, it’s still everywhere and it’s with the people you hang out with and when you take your kids outside, it’s outside.*

(Tasha, Women’s Center 1)

#### 3.1.4. Sense of Control Amid Chaos 

All of the women in this study described experiencing economic and social disadvantages in their lives on various levels. Their experiences of intersecting socially marginalized identities and inequities often left them feeling isolated and defeated. Women stated that smoking gave them a sense of control because they felt overwhelmed or helpless in the face of the myriad challenges of trauma, poverty, substance use recovery, and mental health issues they were confronting. For some, smoking was perceived as exercising control over some aspect of their lives:

*I feel like sometimes I need an external source of control, I can’t control how I feel about myself or what’s going on in my mind, or my anxiety, or the things around me… smoking cigarettes gives me a sense of control… When you struggle with mental illness and depression and anxiety and the stuff that you’ve experienced, when you smoke... I’m doing something to my body that I can control… I can’t control what is going on inside, but I can control what I do to my body. And that kind of makes the craziness in my head isolated to one location.*

(Rose, Women’s Center 1)

### 3.2. Challenges to Finding Better Coping Methods

#### 3.2.1. “Can’t Quit Everything”

A few women resisted having to give up tobacco products, i.e., cigarettes and e-cigarettes, in addition to the various substances for which they sought recovery. Some felt they needed to “hold onto smoking,” to help them navigate the turmoil of relinquishing their drug(s) of choice; they could not “quit everything.” Unfortunately, they, as well as some clinicians, believed that quitting smoking could jeopardize individuals in their substance use recovery, resulting in relapse:

*A lot of people are coming from really extreme backgrounds, like drug use basically. They’re still recovering, but cigarettes are helping them… because that is something that is helping them get through their recovery.*

(Tracy, Women’s Center 2)

As it is common that many staff members at SUTCs have themselves recovered from SUDs and currently smoked, some clinicians worried that quitting smoking would cause colleagues to relapse:

*We have to be mindful that the majority of individuals that are employed here are recovering addicts. And that cigarettes helped them through quitting their drug of choice. So now getting rid of everything, is going to bring up emotions and they could relapse.*

(Sue, Counselor, SUTC1)

#### 3.2.2. Addictive Mentality

Women described themselves as being “addicts,” and “addictive” in nature, substituting food, sweets, Netflix binging, and smoking for their drug of choice to distract themselves when they felt stressed. As with their substance use, these substitute activities were indulged in obsessively, and to excess, where women felt they got “sucked in.” Being addictive entailed being selfish, self-destructive, and reckless:

*I’m not going to sit here and B.S. [smoking] is bad for you. It’s deadly. And the thing is some of us, we get touchy and feely about not being able to smoke cigarettes because we’re addictive. Because we like it and get defensive as cigarette smokers, because we want what we want. But that’s when addiction plays its part, is the selfishness. We will put anything in front of us to defend it, no matter how bad it is for us. It’s a good idea we’re not allowed to smoke here, so that we can find better coping methods.*

(Alma, SUTC1)

#### 3.2.3. Center Support/Attitudes Concerning Going Tobacco-Free

Center support and attitudes regarding transitioning into becoming a tobacco-free environment were mixed, varying according to the individual center. In most partnering centers, clinicians embraced the TTTF program as an opportunity to further assist their clients with rebuilding their lives and their health through a holistic approach to recovery from various harmful substances:

*We’re about the whole woman and her family and how everyone is affected by cigarette smoke… now clients are able to utilize new coping skills instead of running out and having a cigarette. For a smoker that’s your main coping skill because you’re still in that addictive behavior. So, from my point of view, it’s [implementing TFW program] the best thing that we’ve ever done.*

(Martha, Counselor, SUTC3)

While all of the centers’ leadership strongly supported the TTTF program, clinicians in some centers, many of whom smoked, were apprehensive about implementation and doubtful of benefits for clients and clinicians, based on a de-valuing of tobacco addiction relative to other substance use addiction:

*I haven’t seen very much of [smoking counseling] happening, all clients’ time is taken up dealing with more pressing matters, more dangerous behaviors to address, more processing trauma they never had a safe space to talk about. I think counselors are more focused on the bigger problems in their life, which parallels how the clients are thinking about it as well. It’s just smoking.*

(Peg, Counselor, SUTC1)

Although mostly limited to one center, indifference towards, or laxity in, fully adopting and enforcing tobacco-free policies for staff and clients was a substantial challenge to program implementation. Clients reported that tobacco-free policies were inconsistently implemented, and were also disregarded by some clinicians:

*Sally: Sometimes you can see them [counselors] standing over there smoking… half of them are still smoking.*

*IML: So how do y’all feel about that?*

*Cindy: Makes me feel bummed, cause I can’t smoke a cigarette. They shouldn’t be able to either. It’s just like y’all are setting the example for us. We in here recovering and some of y’all have already went through y’all recoveries. So y’all should be showing a prime example of what recovery looks like.*

(SUTC1)

Likewise, our analysis uncovered that widespread, longstanding, and entrenched misconceptions regarding treating substance and tobacco use simultaneously in SUTCs hindered addressing of tobacco dependence:

*My thinking was when I was in treatment, a few years ago, there was no attempt where I was, to address that [smoking] addiction… It doesn’t seem to be an issue for the treatment team to address it here. If the treatment team isn’t addressing it, then you don’t think it’s a big deal.*

(Marla, Recovery coach, SUTC1)

#### 3.2.4. Smoking Culture and Easy Access 

Women’s exposure to different environments where smoking was an accepted norm was a substantial challenge to quitting or maintaining abstinence. These environments included their family and social networks, and unfortunately, some clinicians in the SUTCs where they were seeking assistance. Also, as women shared similar backgrounds and experiences and belonged to groups that experience social and economic disadvantage, in which smoking was an accepted social norm, they naturally reinforced this behavior amongst themselves. While most of our program partners were committed to changing attitudes towards treating tobacco addiction in their centers, they acknowledged that a permissive attitude towards smoking was widespread and entrenched among SUTCs and their clinicians:

*There was a lot of resistance, mainly from other counselors, to actually implementing tobacco treatment into their regular treatment plan. I think the main buy-in was getting staff to accept the idea that nicotine is a drug. Because for such a long-time smoking was accepted in the recovery community; in all the NA and AA groups everybody smoked from the time they started in the 1930s. So, that was the biggest problem that we had; was really getting buy-in from the staff.*

(Martha, Counselor, SUTC3)

Women in residential programs were apprehensive about their likelihood of relapsing regarding smoking, once they left treatment and were exposed to the stresses of daily life and other smokers and had easy access to cigarettes. These women expressed appreciating a TFW policy that banned tobacco use at their center because it kept them from smoking:

*I had stopped smoking for two years and got back with my children’s’ father, who smoked, and from the stress and anxiety that he brought to my life that’s why I started back smoking. At that time, I didn’t know how to cope with my emotions and everything… so, I’m worried because it’s so much easier when it’s legal and it’s everywhere. There are no consequences like with my kids, or jail. I just feel like it’s going to be my biggest issue, I’m not worried about the drugs, but more cigarettes.*

(Juana, SUTC2)

### 3.3. Addressing Underlying Conditions: Building Inner and Outer Supportive Environments 

As clients undergoing treatment for mental health and/or SUDs, many of whom had sought treatment repeatedly, these women were aware of the importance, as well as the challenges, of finding effective, healthy coping strategies as alternatives to substance and tobacco use in response to stress. Learning wholesome ways to ease stress was emphasized as vital to treating their addiction to smoking, and any other substance. They described what they needed to quit smoking based on their experience, which included building a supportive environment focused on learning healthy ways of coping with stress through educational and supportive smoking cessation counseling, peer support, self-awareness, self-compassion, physical exercise, and NRT. 

#### 3.3.1. Smoking Counseling and Peer Support Vital 

Clients and clinicians alike emphasized that smoking counseling support was vital to quitting as it served the dual purpose of supporting women by educating them on the tools they needed to quit and by developing the self-knowledge and self-compassion to move past their tobacco addiction, and was a step towards addressing the psychosocial conditions underlying their stress. Women reported they preferred group smoking cessation counseling, as peer group support was an important reinforcement in quitting. Clients and clinicians also stressed that quitting any addiction, to drugs or tobacco, entails the same process of re-evaluating and making healthy choices in life. For clients, getting support from peers was an important part of the recovery process:

*At first, when I was unable to rationalize and I was angry, I had a lot of self-hate. Along with coping mechanisms and how bad it is for your health, and other things I’ve learned, is part of the reason I’m able to not smoke. I’m not as depressed, I’m not as angry, I go and do other things, like exercise, that help me feel better inside... and the NRT has helped a lot. I don’t think about it as much because I can actually deal with my problems and not mask them with a cigarette.*

(Kayla, SUTC2)

Getting support from peers was also an important part of the recovery process:

*I was never big on asking for help. I was very ‘do it myself.’ But the more sober I get, the more recovery in therapy I get, the more I’m realizing asking for help with something that’s beneficial, it’s not something to be ashamed of. Doing something together is helpful for everybody. Team effort is better because we all want to better ourselves.*

(Chloe, Women’s Center 1)

Clinicians also stressed the value of the support provided via smoking cessation groups and individual counseling:

*It’d be helpful to be in a group simply because of the education provided and the opportunity to talk about the times when they get to a place where they can be aware of when they want that cigarette of what’s going on with them. When I quit, I found myself using a lot of the tools that I’d learned while I was in treatment and it worked for me to quit the cigarettes… what also helped me was the opportunity to talk to people, to have that person I could call and say—Hey, this is where I’m at, this is what I’m going through.*

(Marla, Recovery coach, SUTC1)

*We do a recovery plan within a week of them coming in; their short-term, long-term goals, and smoking is part of that, and it’s amended as they progress in the program. I see a change from day 1 to 90, they acquire more coping skills.*

(Meg, counselor, SUTC2)

#### 3.3.2. Self-Awareness/Self-Compassion

Women reported that through their treatment experience they had learned that developing self-awareness and self-compassion was crucial to a successful recovery. They acknowledged that within their self-described unwholesome social and family backgrounds, substance and tobacco use and other self-harming behaviors were often socially acceptable, which left them ill-equipped to overcome these addictions. Many women felt that it was only through the process of getting to know and understand themselves, their motivations, and the role of addiction in their lives, from a perspective of understanding and self-compassion, rather than judgment, that they could overcome these behaviors.

*So, when you’re high stress, you go to your default setting which isn’t good considering where you come from… for 21 years of my life, it was not good, but I’ve been noticing the more I work on loving and taking care of myself and validating my wounds, I lose those habits without trying. But when I’ve tried to purposefully let go of habits, I relapse and then I relapse, and then I hate myself more, which is a core root problem.*

(Holly, Women’s Center 1)

Through their process of recovery, some women had developed self-awareness regarding their addictive behavior and were learning more about the workings of addiction:

*I’m a recovering alcoholic but I’ll still get these ‘fiends’ for something and cigarettes is what I had. But those ‘fiends’ for something that’ll ‘Ah,’ just relax me, I found even after I smoked that feeling was just stronger, it’s not giving that relaxing feeling I want. I feel if I just remove the dang cigarette that feeling is not as strong… I’m finding that when I quit [smoking] altogether, just like the alcohol, that ‘fiend’ isn’t there anymore.*

(Zoe, Women’s Center 2)

Additionally, many women noted that for them, agency was primary to quitting. Just as with any other substance use recovery, a successful quit attempt was predicated upon their deciding for themselves to quit smoking. No one else can make that decision for an individual; recovery is fueled by internal motivation:

*It was my choice this time that made the big difference. All the times before I was too broke, couldn’t afford it, or the doctor said I needed to quit. This time, I wanted it and it’s been much easier. It’s still not easy, but it’s easier than the other times.*

(Carla, Women’s Center 2)

In contrast, some women rebelled against being put in a position of not having the option to choose, given a center’s adoption of a TFW policy, where they had to quit smoking.

#### 3.3.3. Connections between Smoking and Substance Use

Women reported noting several connections between smoking and substance use, including simultaneously initiating drug or alcohol and tobacco use, increasing their smoking while using their drug of choice, or increasing their tobacco use after quitting their substance use:

*I started smoking when I started shooting dope and got into my addiction. Because the nicotine from the cigarette kept the high going, and it kept me from stressing.*

(LaToya, SUTC1)

Some women viewed tobacco use as another addiction like drug or alcohol addiction and felt that quitting smoking was part of the process of overcoming addiction, as addictive behaviors had the same origins. Likewise, they recognized that smoking triggered substance use for them and saw the value of quitting both at once:

*I think it’s really valuable to quit, that’s one of the last parts of the addiction, still holding on to that last addiction, it triggers my DOC [drug of choice] every time I smoke a cigarette, they go hand-in-hand. I feel like finally releasing that from my life, I’ll be free of it.*

(Kay, Women’s Center 2)

### 3.4. Sustaining Support: TTTF Program Experiences

#### 3.4.1. TFW Program an Opportunity

Most participants from both stakeholder groups valued the opportunities and resources provided by TTTF to assist and sustain clients in quitting smoking. In particular, women credited center adoption of a 100% tobacco-free policy as vital to helping them quit smoking:

*The thing that’s motivated me to stop smoking is, number one: I’m not allowed to smoke.*

(Laura, SUTC3)

*I have been through treatment way too many times and I never gave up cigarettes and I always end up smoking crack… I’m going try something different this time I’m giving up the cigarettes too. I’m glad for it [TTTF] because it’s easier for me to stop smoking in an environment like this. I know I wouldn’t have stopped smoking if I hadn’t been here. Cause I’d still be smoking crack, I’d still be drinking, smoking weed, and cigarettes.*

(LaToya, SUTC1)

*I am glad I’m here because they don’t permit smoking on the premises, otherwise I don’t think I’d be able to stop. I feel a lot better about my health and that I stopped smoking.*

(Donna, SUTC2)

Clinicians valued the TFW program doubly: for helping women quit smoking and for the support that smoking cessation provided to their clients’ successful substance use abstinence:

*A lot of clients are heavy smokers, without this program I don’t think they’d stay, people leave because they can’t smoke, that’s their excuse. The NRT’s really helped women with their anxiety, stress, whatever they feel the cigarettes were helping them with, giving them treatment. Cigarettes is key to relapse in drug abuse, this program helps them with their triggers and offers them everything we can to successfully recover. This program is one of the biggest helpers in determining how successful they’re going to be when they leave.*

(Anita, Counselor, SUTC2)

Although a few clinicians and clients were not supportive of the program, most welcomed it and recognized that quitting smoking could assist women in their substance use recovery. While women recognized that readiness to quit was important, even women who reported they were not ready to quit, expressed appreciation for the smoking cessation services provided by the TFW program to those wanting to quit:

*I appreciate the program, it’s wonderful for someone that’s ready to quit. [Counselors] are accommodating people that are ready to quit, but it’s a waste of time for those not ready to quit. But I believe it’s very worthwhile. They’re doing the best they can now.*

(Karen, SUTC3)

#### 3.4.2. NRT Facilitates Quitting

In keeping with the clinical guideline recommendations of the U.S. Preventive Services Task Force [56] we do not recommend NRT for pregnant women; that decision should be made by the woman and her physician. A few women reported either disliking the taste of the NRT lozenges or gum, or experiencing side-effects such as dizziness, nausea, or localized rashes from using the patch, rendering NRT use infeasible. However, most women reported no difficulties with the NRT and that it had provided the support they needed to quit smoking:

*I am thankful, very thankful, for y’all’s help. I am ecstatic. I’ll be stressed out and take that gum and I’m settled down. It’s a miracle that I have been able to do it [quit smoking].*

(Lucy, SUTC2)

*This is the first time I’ve tried NRT to quit smoking. Every other time I’ve tried cold turkey and it hasn’t ever stuck. My hopes are high that, along with the education I’m getting about addiction, might help me abstain from smoking more successfully than in the past. Even though it wasn’t my choice initially, I don’t want to continue smoking. So, I’m optimistic about being able to do it this time.*

(Joy, SUTC3)

*[NRT] is really the only thing that’s helped. I think it’s the patch with the lozenge because it’s an oral fixation, smoking is something in your mouth all the time.*

(Laura, SUTC3)

#### 3.4.3. Non-Judgmental Approach and Support

Women and clinicians alike stressed the importance of using non-judgmental approaches in addressing recovery from substance use and nicotine addiction. Many women had been in recovery multiple times and had, unfortunately, internalized some of these shaming and condemning approaches from counselors; they knew first-hand how detrimental and unsuccessful such strategies were in overcoming addiction:

*Every time I relapsed, and felt ashamed and bad, I’d tell myself ‘It’s okay, try again.’ I just kept getting up and getting up and failing and, eventually, I did stop. The big thing that helped me was having that mentality... and if there was openness about that [with counselors] without the condemnation of ‘Don’t do it,’ is a great thing... guilt and shame have kept me stuck in those cycles of going to your coping mechanisms [cigarettes, drugs]. If you quit smoking, you’re probably going to create another negative coping mechanism because it’s not the cigarette itself. You really have to deal with the guilt and shame, that is the hardest part… so focus on self-love and healing the individual and those bad side effects will slowly disappear on their own because they won’t need that negative coping mechanism anymore.*

(Holly, Women’s Center 1)

Clinicians were also aware of how damaging judgmental and shaming attitudes and methods could be to clients and their recovery and were sensitive to not present the TFW program with that intent:

*An effect of having a non-smoking facility I wish could be changed somehow… avoiding creating that feeling that I’m going to be judged by my counselor if I say I’m not ready to quit. Because, from my experience replacing unhealthy coping with healthy coping, an essential part of that process was detaching the judgment that unhealthy coping is bad or shameful… you give them support until they’re ready to replace it with healthy coping.*

(Peg, Counselor, SUTC1)

*That’s one thing I’m concerned about, I don’t want that tool [the TFW policy] to be used to judge or police them... I can see that being pretty counterproductive.*

(Ivy, Counselor, SUTC1)

### 3.5. Specific Program Recommendations

Clients’ and clinicians’ specific recommendations on what women needed to support them in quitting smoking was elicited pre-implementation and used to tailor the TTTF program to their needs and preferences. Specific program recommendations (Table 3) consisted of varied smoking cessation strategies, i.e., medical, or behavioral interventions or supports viewed as most valuable in assisting in smoking cessation. Recommendations included: (1) use of varied NRT products, i.e., lozenges, gum, and patches, as clients reported side-effects with only a particular product; (2) weekly smoking cessation group counseling to provide peer support in quitting; (3) provision of exercise programs or facilities to help alleviate stress; (4) providing women in centers that still allowed smoking with separate smoke-free living environments; (5) supplying clients’ family members with NRT to help them stay smoke-free; and (6) providing clients with the support and education to understand and overcome associations between stress and smoking.

## 4. Discussion

Many studies on smoking cessation interventions among women with SUDs have focused specifically on pregnant women [57,58], whereas few have investigated tobacco use recovery efforts among the larger population of women with SUDs [5,6]. Our study contributes to and expands upon a limited body of literature on developing smoking interventions tailored to women with SUDs experiencing disadvantage. To our knowledge, this is the first qualitative study of women with SUDs experiencing disadvantage focused on participants’ experiences of a smoking cessation intervention that was tailored to the social context of their smoking and their cessation needs in collaboration with women in multiple settings. Findings are framed within the intersecting psychosocial and structural inequities that constrain the lives and choices of women experiencing disadvantage and describe the meanings they attribute to smoking and their experiences and recommendations regarding the TTTF program to inform the tailoring of effective smoking cessation interventions based on their needs and preferences. Study findings attest to the fact that in spite of the multiple challenges and disadvantages these women face, they are willing and capable of reducing or quitting smoking when they are provided respectful, compassionate, and supportive care and cessation services that are responsive to their circumstances. As such, this study contributes to a small body of research [5,6,59] which recognizes that the challenges and complexities of addressing smoking cessation among women with SUDs should compel rather than deter researchers from engaging in interventions tailored to their circumstances given the high rate of smoking among this population and their dire need for assistance in quitting.

### 4.1. Responding to the Social Context of Smoking and Quitting

Women described smoking as primarily serving an adaptive function, as previously noted by others [13,60], in this case, to gain social acceptance, to adapt to the stress of living under multiple disadvantages including substance use and mental health disorders, poverty and social deprivation, unstable housing, single motherhood and histories of violence, abuse, and trauma and afforded them a sense of control amidst the chaos of their lives. These multiple psychosocial stressors can result in high levels of perceived stress, which has been associated with a higher prevalence of smoking [61], particularly among women [8]. However, abundant research also shows that nicotine has been associated with increasing stress [24,62,63], which in turn drives smokers to smoke even more, trapping them in an endless cycle of addiction. Withdrawal symptoms of nicotine include anxiety, irritability, and depression, which are reliably alleviated by smoking, leading smokers to conclude that smoking provides psychological benefits, when smoking and nicotine withdrawal is what initially caused these psychological disturbances [24]. As such, misattributing stress relief with smoking, which is also promoted in tobacco advertising [7], can only worsen these symptoms of stress. Supporting cessation among these women also necessitates correcting this misattribution by decoupling smoking from stress relief. Overcoming the cravings and withdrawal symptoms of recovering from any addiction is a complex and stressful process. Behavioral health clinicians do not question the value of assisting women to overcome their substance use addiction, yet, as study participants reported, they often fail to treat tobacco as a harmful addiction, even though high mortality rates among those with alcohol and SUDs are attributed to tobacco-related diseases rather than alcohol or drug use [64]—with more than 50% of those in treatment dying from smoking-related diseases [25]. 

Through their own experience, some of the women in this study became aware of how smoking only increased their experience of stress; smoking did not satisfy their craving, or “fiend,” but rather increased it and did not make them feel more relaxed or satisfied as promised. Such experiential insights into the relationship between stress and smoking provide clinicians with a valuable opportunity to meaningfully address smoking cessation and to correct this misattribution. Thus, supporting women to relinquish a behavior that is physically unhealthy to them and their families, that threatens their psychological wellbeing by increasing anxiety and depression, and that keeps them trapped in a vicious cycle of nicotine addiction and increased probability of SUD relapse [65]. Research indicates that former smokers will reap the psychological benefits of quitting by around 3 weeks, due to the abating of withdrawal symptoms [66]. Moreover, even within these treatment and women’s center—either before they adopted a TFW policy or for those that did not—clients and clinicians reported that smoking continued to be the main social activity for women, which led those who had quit or were non-smokers to take up smoking. Unfortunately, the failure to replace smoking within these centers with another healthy social activity (e.g., group “walks” for physical activity) represents a missed opportunity for smoking cessation and recovery in general, and for correcting the misattribution of stress relief with smoking, as it reinforces smoking as the requisite social currency, the means of “relaxing” and socializing [57]. This practice can also increase the internal conflicts women experience around smoking in wanting to socialize while also feeling shame and compelled to quit for their children [67]. Stigmatization for smoking among pregnant women or those with small children is high [68]. Mothers within this study shamed themselves and others for smoking, particularly during pregnancy; for many it was only during pregnancy that they had successfully quit, although post-partum relapse rates are high [69,70,71] and at least partially attributable to negative affect/stress and low perceived agency (i.e., self-efficacy/self-confidence), which may be common among women facing myriad disadvantages. However, a number of smoking cessation interventions have focused on women with SUDs’ increased motivation to quit while pregnant for the sake of their child’s health as an opportunity to enlist them in successfully giving up smoking [59]. Framing smoking cessation negatively, where guilt, shame, or stigmatization is used to motivate already stigmatized female smokers to relinquish a behavior that affords them pleasure not only fails to support quitting, but increases resolve to continue smoking [68], even though women also perceived smoking as self-destructive. The fact that some women continued to smoke under these conflicting and difficult circumstances underscores the complexities of their tobacco addiction and challenges to quitting. 

Women also stressed that working with compassionate counselors who did not shame or blame them was essential [60] as it was through their counseling work that they had learned the importance of self-awareness and self-compassion in the recovery process. Many women reported that emphasizing shame and guilt, whether arising from self or others, only kept them in the vicious cycle of addiction. For other women, agency in quitting was primary; they attributed successfully quitting to having made the decision themselves, rather than being told, to quit [72]. These women benefited from motivational interviewing, which recognizes that readiness to change habits results from inner change combined with external opportunities that align with a time and pace appropriate to the individual [73,74]. Many reported smoking and substance use triggered each other [75]; ample research documents the association between smoking and substance use [13,14,15]. Engaging in smoking and substance use was so inextricably connected for some women that they reported feeling that giving up smoking would free them from their substance addiction. 

The women within this study faced various external and internal challenges to treating their tobacco dependence that required dedicated support that, unfortunately, was not always available, given the prevalent misconception that clients could not quit smoking while in recovery and center attitudes on becoming tobacco-free, which could either facilitate of hinder client smoking. Some clinicians supported the notion that treating tobacco and substance use addiction simultaneously could jeopardize SUD recovery; attempting to recover from both would stress clients excessively, resulting in their SUD relapse and undervalued tobacco addiction as a problem; which has been recognized as a serious barrier to treating tobacco dependence within these settings [63]. In spite of significant research demonstrating that, on the contrary, quitting smoking increases abstinence from alcohol and other substances by 25% [18], decreases anxiety [22], and increases overall psychological quality of life [24], this misconception regarding smoking and substance use treatment is entrenched among behavioral health clinicians and communicated to clients. These attitudes then become internalized by clients, to become additional internal challenges to quitting augmented by the absence of clinician support in addressing smoking. Other studies confirm clients and clinicians in SUTCs often do not support women in quitting smoking while in recovery; in fact, clinicians encourage women to smoke as a means of “pacifying” them [6]. The failure of treatment providers to conceptualize and frame tobacco use disorder as a chronic and relapsing disease in need of recovery advocacy and support—similar to other addictions—can disenfranchise clients from getting the care they need in behavioral health treatment settings [76]. However, most of our program partners were dedicated to changing the permissive “culture of smoking” traditionally associated with behavioral health and addiction treatment centers.

Key to addressing smoking was learning alternative, healthy coping strategies to replace the adaptive functions of smoking in their lives through supportive and educational smoking cessation counseling that included physical activity, relying on peer support, and using NRT to relieve nicotine withdrawal symptoms linked to perceived stress [8]. Physical activity has been demonstrated to acutely reduce cigarette craving [77,78]. A recent tobacco treatment intervention for women in a SUTC based on physical activity and smoking cessation counseling resulted in successfully reducing nicotine dependence and smoking among participants [5]. Likewise, both of our stakeholder groups emphasized peer group smoking cessation counseling was preferable, as peer support was also essential [42]. Many clinicians had recovered from SUDs and smoking themselves and knew first-hand how essential social support interventions were to their successful recovery. Cessation interventions that increase social support are recognized as successfully promoting cessation among women experiencing socioeconomical disadvantages [79], and are especially relevant because: (1) these women live in environments where smoking is socially accepted and may need additional social support while tackling cessation [13]; and (2) economic realities mean they must rely on free psychosocial support offered by community health programs or state Quitlines for quitting assistance [41]. Women were concerned they would resume smoking once they returned to their “normal” life (i.e., were discharged from the residential setting) where smoking was an accepted social norm [41]. In fact, some were more concerned about relapsing regarding tobacco than illicit drug use, given the legality and accessibility of tobacco, and therefore were grateful for receiving treatment in a smoke-free environment that supported them in their efforts to quit. This finding corroborates the value of TFW policies in promoting successful quit attempts among those experiencing disadvantages and as an important part of their tobacco recovery [80]. Here again, these women require additional support to continue to be smoke-free, such as distributing NRT to family members as well as clients [32], whenever feasible, as encouraged by TTTF. Adoption of supportive community-level interventions, such as tobacco-free policies and smoke-free homes can provide important additional social supports to women and their families to remain smoke-free once they have left treatment centers [81,82,83].

### 4.2. TTTF Program Experiences

As clients and clinicians collaborated with researchers to tailor the TTTF program in these SUTCs to the needs and preferences of the women, it is important to understand their program experiences regarding implementation for improvement of future women-centered smoking cessation interventions for women with SUDs experiencing disadvantages. Overall, clinicians’ and clients’ experiences regarding TTTF were largely positive. Even those clients who were either not interested in quitting or rebelled against not having a choice about quitting while in recovery saw TTTF as an opportunity to quit smoking for those interested in doing so. In fact, many women said they were grateful for the TFW policy that banned smoking while they were in recovery, as they never would have quit otherwise, and they recognized that quitting supported their SUD recovery. A number of these women had been in recovery repeatedly without ever having been offered the chance to also quit smoking, and they had all relapsed [65]. For these women, the opportunity offered by TTTF to quit all of their addictions at once made sense to them, as substance and tobacco use go hand-in-hand. Women were happy they had quit, reporting feeling better, healthier, and that their children were grateful they had stopped smoking. Clients and clinicians stated that the supports they received from TTTF, i.e., NRT, supportive and educational smoking cessation counseling and compassionate motivational support, had helped clients to quit smoking. Additionally, in response to clinicians’ and clients’ requests, we developed materials and strategies to facilitate program implementation and community outreach, based on collaboration with these stakeholders, e.g., educational brochures on the hazards of second-hand smoke and smoking for pregnant women. It is important to note that although all of the SUTCs had adopted a 100% tobacco-free policy within their centers’ parameters, women could sneak a smoke while off-campus attending referred treatment, whereas the women’s center did have designated smoking areas. As such, even though women in the SUTCs were enrolled in a tobacco-free program, most could exercise their choice to smoke or to quit smoking.

### 4.3. TTTF Program Recommendations

Clients’ and clinicians’ specific recommendations for TTTF program implementation included: offering clients various types of NRT, i.e., gum, patches and lozenges; holding regular smoking cessation counseling groups; options for engaging in physical activity; housing women who did not want to smoke together in their own smoke-free environment; providing NRT to clients’ families; integrating smoking cessation into SUD counseling or 12-step program; and adopting non-judgmental approaches and support. Each of the different SUTCs and women’s centers that participated in the TTTF program were unique and had leeway on implementation of program adaptations to their center according to their needs and resources. Regarding participants’ recommendations, some centers offered these resources to clients, others did not. For example, although recommended to do so, not all centers held smoking cessation counseling groups for clients. 

However, participants’ experiences and recommendations on what they need to quit smoking are supported by the TTTF program and have been successfully implemented by our program partners [32,36,37], which is aligned with the recommended components of a women-centered approach to tobacco cessation [13,60]. Researchers advocate that smoking cessation interventions for women experiencing disadvantages should: (1) be tailored to women’s needs; (2) focus on building confidence and motivation; (3) incorporate social justice issues; and (4) adopt a holistic and comprehensive approach [13]. TTTF is allied with these recommendations. Firstly, in conducting formative research to adapt the program to women’s needs, which included counselors drawing up individualized recovery and quit plans for women, TTTF was women-tailored. Secondly, clinicians were trained in using non-judgmental approaches to address client tobacco use, including motivational interviewing and the 5A’s (Ask, Advise, Assess, Assist, Arrange). Thirdly, sensitivity to social justice issues and not increasing the stigmatization of these women experiencing vulnerabilities guided program design and was seen, for example, in educational programming that emphasized the tobacco companies’ unscrupulous marketing practices. While denormalization strategies, which attempt to influence social norms as a means of supporting smoking cessation, can contribute to stigmatization [72], TTTF relied on norms-related approaches focused on changing smoking behavior that did not increase inequality and stigmatization [3,84]. We adopted a social norms marketing strategy, which is based on the idea that perceived social norms influence our behavior; however, individuals’ understandings of social norms are based on perceptions of certain behaviors, which may be incorrect [84].

Through our training of clinicians, and clinicians’ educational smoking cessation counseling for clients, we sought to correct the misattribution of stress relief to smoking and misperceptions regarding smoking and SUDs, such as the beliefs that smoking cessation jeopardizes SUD recovery and that those with SUDs are unable, or are not motivated, to quit smoking. As these misperceptions contribute to smoking behavior, correcting them served as a crucial strategy for achieving smoking cessation through correction of a biased inequality in the choices faced by these women experiencing disadvantages. That is, those within groups experiencing disadvantages that have a higher prevalence of smoking can perceive norms through a misleading lens that could increase their likelihood of smoking [3]. Likewise, a tobacco industry denormalization strategy was included in our trainings for clinicians, in which, rather than focusing on denormalizing and vilifying tobacco use among women, the aim was to alter perceptions of the tobacco industry, revealing the fraudulent, unethical, and manipulative tactics used to target groups facing disadvantages [84]. As the tobacco industry is also complicit via its targeted campaigns to distort social norms regarding smoking among those living with disadvantages, both of these norms-related strategies are equitable and non-stigmatizing, as they shift the blame for smoking away from the individual smoker to the context of smoking and to the tobacco industry. Lastly, the TTTF program adopts a comprehensive approach, targeting system-level changes (organization, community, clinician, client), and adopts an integrated approach to treating mental health, SUDs, and tobacco use, given the co-occurring disorders experienced by these women confronting multiple disadvantages. Through our program, women were provided with motivational support, behavioral counseling, and pharmacological support, e.g., varied NRT, that assisted them with nicotine withdrawal, and which they reported facilitated their quitting, in keeping with best practices for smoking cessation [85]. 

### 4.4. Study Limitations and Future Research 

As a study focused on contributing to the tailoring of smoking cessation interventions to women experiencing various disadvantages, the applicability of findings may be limited. Pre- and post-implementation focus groups were conducted with different clients (e.g., due to 90-day residential programs), which limited the extent to which the same clients could comment on pre- and post-implementation center changes. However, findings were consistent with and support those of other studies focused on women with SUDs experiencing social disadvantages [6,13,60]. A strength of this study is that key stakeholders, clinicians, and clients were enlisted as collaborators in adapting the program to women’s needs and preferences. Additionally, program detractors, as well as program supporters, were purposefully selected to participate in focus groups, to minimize social desirability bias. Given the centrality of addressing these women’s high level of stress and their misattribution of stress relief with smoking, intervention studies are needed that focus on investigating the effects of this misperception among women in treatment for SUDs. The increasingly wide inequalities between those living with advantage and those living with disadvantage can only lead to further stigmatization of smokers experiencing disadvantage, necessitating more research that targets social inequities and contextualizes health behaviors. Finally, as female smokers experiencing disadvantage live under the burden of multiple social inequalities, targeting specific health behaviors such as smoking may not be sufficient. More comprehensive smoking cessation projects are needed that target the multi-level factors, rather than simply the individual factors, that together create the conditions of vulnerability contributing to smoking among these women. The complexity of addressing smoking cessation among women undergoing disadvantage requires research that provides evidence of “best practices” that have proven effective in addressing tobacco dependency among this group [70,74,86]. 

## 5. Conclusions

Study findings demonstrate how implementing a comprehensive, multi-level smoking cessation intervention informed by the meanings of smoking for women experiencing disadvantage in treatment for SUDs and their needs and preferences regarding quitting, can better respond to the complexities of tobacco dependence among this group to enhance supportive and effective cessation. Addressing tobacco reduction or cessation among women with SUDs is challenging and requires commitment, as these women require dedicated, focused, and contextualized support, which most critically starts with supporting and re-educating clinicians in using non-stigmatizing and equitable approaches to tobacco cessation. Assisting women in learning healthy ways of coping with stress as well as correcting clinicians’ and clients’ prevalent misconceptions that support smoking—e.g., smoking relieves stress, and quitting smoking jeopardizes SUD recovery—were crucial to helping them quit. The components women identified as necessary to help them quit—the provision of NRT, and non-shaming, compassionate, and supportive strategies that assisted them in developing self-awareness and self-compassion and understanding of the connections between their drug and tobacco use—were aligned with the TTTF program that responded to the context of smoking within their lives. Clients and clinicians alike reported that tailored interventions should include non-judgmental approaches, use of varied NRT, dedicated smoking cessation counseling and peer support, physical activity, and access to tobacco-free environments, including the adoption of a TFW policy, to facilitate tobacco use recovery. Study findings demonstrate how to adapt a TFW program to be responsive to the intersecting social inequities that constrain the lives and choices of these women, through tailored interventions and strategies that promote equity in tobacco control policies and decrease further stigmatization of women smokers experiencing disadvantage. These findings present an effective model for treating tobacco dependence tailored to the needs and preferences of women experiencing disadvantage that they reported was successful in assisting them to quit smoking while in SUD recovery. TTTF serves as a model of an effective comprehensive TFW program that can be tailored to the needs and populations of community centers interested in treating tobacco dependence.

## Figures and Tables

**Table 1:** Characteristics of participating centers.

Center	# Clinics	# Clinical Staff	% Smokers	Total Annual Unique Clients	Total Annual Contacts	Residential/ Outpatient
SUTC1	2	131	74	1004	1004	Both
SUTC2	1	22	75	1135	1670	Both
SUTC3	1	45	66	1216	22,052	Both
Women’s Center	2	11	65	77	77	Both

Note: SUTC = substance use treatment center.

**Table 2:** Demographic characteristics of center clients.

Client Characteristics	SUTC1	SUTC2	SUTC3	Women’s Center
Unemployed	94%	86%	84%	100%
Housing-no stable or permanent housing	89%	50%	87%	100%
Prior criminal justice system history	64%	74%	64%	33%
CPS active status	43%	32%	45%	33%
Domestic violence/ abuse/trauma history	95%	88%	82%	66%
Substance use disorder	100%	100%	100%	27%
Psychiatric disorder (co-occurring)	78%	54%	52%	11.8%
At or below poverty level	100%	64%	73%	100%

Note: SUTC = substance use treatment center; CPS = Child Protective Services.

**Table 3:** Specific program recommendations from clients and clinicians.

Participant Quotes	
Varied NRT	*If we could get the gum in here it would help me a lot… because I cannot wear the patch. It makes me sicker than a dog. I’ve tried lozenges and they are just too strong. I could only suck on it for a little bit then had to get rid of it because it was too strong.* (Kat, SUTC1)
Smoking cessation group	*It’d be good if we had a smoking group, once a week, that would really help us.* (LaToya, SUTC1)
Exercise options	*I wish we had a place so we could work out vs. smoking. I know that sounds stupid, but if we had another place to get rid of the stress, and there’s not that here.* (Amy, SUTC1)
Smoke-free living environment	*Brenda: yeah, if you stick those people who are at the same level, on wanting to quit together and have them all be neighbors… in their non-smoking neighborhoods. That could be beneficial, they could support each other. Because you don’t want to stick somebody who’s trying to quit, right in the middle of all these people who are smoking, it’ll make it 10 times harder for you to quit.* *Caitlyn: Yeah, it’s an incentive to take care of yourself and your kids…* (Women’s Center 1)
NRT for family	*Is there help for our families? Like when we get out of here, it would be great to be able to get some sort of assistance or discounts, as far as continuing the NRT, to help them also.* (Lucy, SUTC3)
Education on link between stress and smoking	*Showing and teaching them consistently how to get past the cravings and the connection between stress and smoking… bringing that attention to the benefits of stopping would really help the ladies.* (Marla, Recovery coach, SUTC1)

Note: SUTC = substance use treatment center; NRT = nicotine replacement therapy; all names are pseudonyms.

## Data Availability

Data available on request due to privacy restrictions. The data presented in this study are available on request from the corresponding author. The data are not publicly available due to agreements with funders.
